# Heterozygous Single Nucleotide Polymorphic Loci in Haploid Gametophytes of *Gracilariopsis lemaneiformis* (Rhodophyta)

**DOI:** 10.3389/fgene.2019.01256

**Published:** 2019-12-06

**Authors:** Yiyi Hu, Zhenghong Sui, Wei Zhou, Jinguo Wang, Minjie Jiang, Haihong Chen, Jingyu Zhang, Wei Zhang, Xiaoqing Feng, Yuehua Lei, Baoheng Xiao, Peng Guo

**Affiliations:** Key Laboratory of Marine Genetics and Breeding (Ocean Univeristy of China), Ministry of Education, Qingdao, China

**Keywords:** *Gracilariopsis lemaneiformis*, haploid gametophyte, single nucleotide polymorphism, heterozygous genotype, whole-genome resequencing

## Abstract

*Gracilariopsis lemaneiformis* is an important commercial macroalga. Whole-genome resequencing was conducted to identify single nucleotide polymorphisms (SNPs) in parental gametophytes and 60 F_1_ gametophytes of *Gp. lemaneiformis* in this study, and 9,989 SNPs located in nonrepetitive sequences were obtained. Among these SNPs, 92.02% of loci were identified as having a heterozygous genotype in at least one gametophyte, and 48.07% of loci had identical heterozygous genotypes in the 62 gametophytes. For each gametophyte, the proportions of homozygous and heterozygous loci ranged between 13.74 and 21.61% (mean of 17.04%) and between 66.36 and 83.59% (mean of 77.12%), respectively. The remainder were missing loci, representing an average 5.84%. Sources of heterozygous SNPs were free of exogenous DNA contamination, cross contamination among individuals, plastid and mitochondrial sequences, chimeras of different thallus parts or different cells, and repetitive sequences. Genotypes of heterozygous SNPs were verified by Sanger sequencing of PCR products and monoclones. Duplications of chromosomal rearrangements in the genome of *Gp. lemaneiformis* might explain the presence of heterozygous SNPs in haploid gametophytes.

## Introduction

*Gracilariopsis lemaneiformis* (Bory de Saint-Vincent) E. Y. Dawson, Acleto & Foldvik is a commercially important macroalga that is mainly utilized for agar extraction and abalone aquaculture (Santelices and Doty, 1989; Mchugh, 1991). The high content of good quality agar produced by *Gp. lemaneiformis* represents 53% of the annual agar output worldwide (Mchugh, 1991). *Gp. lemaneiformis* is rich in glutamic acid and taurine, which can enhance the flavor of abalone and promote its feed intake (Chopin et al., 2001; Fei, 2004; Neori et al., 2004; Lu et al., 2011). In addition, *Gp. lemaneiformis* can effectively reduce the contents of N and P in the seawater and plays a role in bioremediation (Fei, 2004).

*Gp. lemaneiformis* is an ideal material for genetic research. Its whole life history can be completed in a laboratory culture (Plastino and de Oliveira, 1988). Mature tetrasporophytes (2n) produce tetraspores (n) through meiosis, and then tetraspores are released and develop into male and female gametophytes (n) (Engel et al., 2010). After fertilization, the zygotes develop into carposporophytes (2n), which release carpospores (2n) through mitosis (Guzmán-Urióstegui et al., 2002). Subsequently, carpospores develop into tetrasporophytes (Yamamoto and Sasaki, 1988). The reproductive modes of *Gp. lemaneiformis* include both sexual and asexual reproduction. In recent years, genetic studies on *Gp. lemaneiformis* have focused on genetic diversity analyses (Wang et al., 2007; Pang et al., 2010; Ding et al., 2012; Hu et al., 2018), functional gene studies (Liu et al., 2016; Liu et al., 2018; Hu et al., 2019), mutation research (Zhang and van der Meer, 2011; Fu et al., 2014), mitochondrial genome (Zhang et al., 2012), chloroplast genome (Du et al., 2016) and genomic studies (Zhou et al., 2013; Sun et al., 2018)

Currently, *Gp. Lemaneiformis* is broadly cultivated in Guangdong, Fujian, Zhejiang, Shandong, and Liaoning Provinces, representing the second most cultivated seaweed in China. Screening for new strains and developing large-scale industrialized cultivation methods are of great significance for the use of this species. Molecular marker-assisted breeding technology can be used for mapping important quantitative trait loci (QTL), allowing the selection of a trait by analyzing molecular markers that are closely linked to the target trait’s loci. It is an effective way to accelerate the breeding process (Ribaut and Hoisington, 1998). Genetic linkage maps play a fundamental role in QTL localization by providing selectable markers for traits selection (Li et al., 2015; Lu et al., 2018; Raman et al., 2018). Due to their relative abundance, high polymorphism rate, stable inheritance, and wide genomic distributions, single nucleotide polymorphism (SNP) markers have been widely applied in molecular marker assisted breeding (Berger et al., 2001; Wicks et al., 2001; Stickney et al., 2002; Chen et al., 2008). The development of SNP markers for breeding have never been conducted in *Gp. lemaneiformis*. With the advent of next-generation sequencing which provide rapid and low-cost methods for genotyping large populations, SNP markers can be obtained using reduced-representation sequencing (Baird et al., 2008; Kerstens et al., 2009; Wang et al., 2012; Poland et al., 2012; Fu et al., 2016) and whole-genome resequencing (Xia et al., 2009; Huang et al., 2009; Lam et al., 2010; Cao et al., 2011).

Because of the industrial importance of the species and the usefulness of SNP markers in species breeding, SNPs were exploited in the parental gametophytes and 60 F_1_ gametophytes of *Gp. lemaneiformis* in this study. If the F_1_ tetrasporophyte (2n) is heterozygous at one SNP locus, F_1_ gametophytes (n) should be homozygous and the segregation ratios of the two genotypes should be 1:1 at this locus. However, we identified abundant heterozygous SNP loci in gametophytes, and we intend to investigate where the heterozygous SNP loci originated. This study promotes investigating the genomic characteristics of *Gp. lemaneiformis*, which will be of great importance in guiding studies on the genomic evolution of marine algae.

## Materials and Methods

### Construction of Mapping Population

The life history of *Gp. lemaneiformis* includes a gametophyte phase, which is equal to the haploid population, and it can be used for genetic linkage mapping. In this study, the female gametophyte ♀6 and male gametophyte ♂9 were produced from Cultivar Lulong No. 1, which is tetrasporophyte strain. F_1_ tetrasporophytes were produced from a cross between ♀6 and ♂9. Then, mature F_1_ tetrasporophytes gave rise to tetraspores, which subsequently developed into F_1_ gametophytes. Whole-genome resequencing was carried out for the two parental gametophytes and 60 F_1_ gametophytes.

### Whole-Genome Resequencing Protocol

Genomic DNA was extracted using a Plant Genomic DNA Kit (Tiangen Biotech, Beijing, China) following the manufacturer’s instructions, and at least 1 μg qualified genomic DNA was used for constructing a whole-genome resequencing library. Genomic DNA was sheared randomly into fragments, and 300–500-bp fragments were purified using gel electrophoresis. The sequencing library was prepared using a TruSeq™ DNA Sample Prep Kit and a TruSeq PE Cluster Kit. The prepared library was sequenced with paired end reads (2 × 150 bp) on an Illumina HiSeq 4000 sequencing platform. The whole-genome resequencing data of 62 gametophytes have been deposited in Short Read Archive (SRA) database with Project number PRJNA574029, under the accession number SRP223151.

Raw sequence reads were processed using custom PERL scripts to separate them into individual fastq format files based on the barcode sequence of each sample. Contamination reads, like those containing adaptors or primers, were identified using SeqPrep (https://github.com/jstjohn/SeqPrep) with the following parameters: “'-q 20 -L 75 -B AGATCGGAAGAGCGTCGTGT -A AGATCGGAAGAGCACACGTC.” Sickle (https://github.com/najoshi/sickle) was applied to perform read data trimming with default parameters to produce clean data (high quality data) in this study. The high quality sequencing reads were aligned to the reference genome sequence using BWA (http://bio-bwa.sourceforge.net/) software in “bwa aln” mode. The genome survey sequencing of *Gp. lemaneiformis* had been conducted previously (Zhou et al., 2013), and the assembled scaffolds were used as reference genome sequences in this study. After removing PCR-duplication reads using SAMtools (http://samtools.sourceforge.net/) software, the sequencing depth and coverage were calculated based on alignments performed by custom PERL scripts.

### SNP Calling

The valid BAM file was used to detect SNPs using the GATK “UnifiedGenotyper” function (http://www.broadinstitute.org/gatk/). Then, variant call format (VCF) files were generated by quality filtering (VariantFiltration with parameters: QD < 2.0 || FS > 60.0 || MQ <40.0 || SOR > 10.0). Furthermore, VCF files were filtered using VCFtools (version 0.1.11; parameters: –minQ 20 –minDP 4).

### Verification of Heterozygous SNP Loci

The SNP loci that had identical heterozygous genotypes in the two parents, were selected to determine whether they belonged to exogenous DNA contamination. Sequences of 400 bp in which a heterozygous SNP locus was located were obtained from reference genome sequences, and served as target sequences. BLASTN in NCBI with default parameters was used for sequence annotation. The SNP loci that had a genotype that was homozygous (or missing) in one parent but heterozygous in the other, were suspected to result from cytoplasmic inheritance. Sequences of 400 bp containing the target SNP were aligned to the complete plastid and mitochondrial sequences.

SNP primers were designed using Primer 6.0 and synthesized by Sangon Biotech (Shanghai, China). The reaction solution for PCR amplification contained 20 ng genomic DNA, 1× PCR buffer, 1.5 mm Mg^2+^, 0.2 mm dNTPs, 0.5 μm of each primer, 0.5 U *Taq* DNA polymerase (Fermentas, Burlington, Canada) and autoclaved distilled water to a total volume of 20 μl. The PCR program performed was as follows: initial denaturation at 94°C for 5 min; 35 cycles of denaturation for 1 min at 94°C, annealing for 1 min at 60°C and extension for 1 min at 72°C; a final extension step for 5 min at 72°C. Sanger sequencing of PCR products and monoclones were completed by TsingKe Biological Technology (Qingdao, China)

### Chimera Testing

To determine whether heterozygous SNPs resulted from chimerism, a single gametophyte of *Gp. lemaneiformis* was cut from the base to the top, obtaining 22 1 cm segments. The segments included nine representative parts: base, middle part, and tip of primary/secondary branch; junctions of primary and secondary branches; junctions of secondary and tertiary branches; tertiary branches. The genomic DNA of every segment was extracted and amplified using primers for SNP locus scaffold1837_730, scaffold7339_502 and scaffold7339_533, and then, the PCR products were analyzed by Sanger sequencing.

### SNP Detection in Single Cells

A gametophyte thallus was used to produce a cell suspension by grinding in liquid nitrogen and filtering. Then, single cells were isolated in a glass capillary tube under a microscope. Genomic DNA of each cell was extracted and amplified using a Single Cell Whole Genome Amplification Kit (Yikon Genomics, Suzhou, China) following the manufacturer’s instructions. Then, heterozygous SNP loci were identified using PCR amplification and Sanger sequencing.

## Results

### Development of SNPs Using Whole-Genome Resequencing Technology

The statistical data of the whole-genome resequencing are listed in **Table S1**. The total data for the female gametophyte parent, male gametophyte parent and the mean of the F_1_ gametophytes were 1.21, 1.36 and 1.62 Gb, respectively. The GC content range per individual was between 45.03 and 52.53%, with a mean of 48.92%, and all three values were in the normal range. The mean Q20 value was greater than 95%, and the mean Q30 value was greater than 91%, revealing the high quality of sequencing. The depth of the female, male gametophyte parents, and the mean of the F_1_ gametophytes, were 12×, 14× and 16×, respectively. Each gametophyte was sequenced to an average 80.15% coverage of the genome. The base distribution and base quality distribution of whole-genome resequencing data were shown in **Figures S1** and **S2**.

The high quality sequencing reads of each individual were aligned to the reference genome sequences, and the mapping results are displayed in **Table S2**. The mapping rate of the female parent was 84.35% and that of the male parent was 49.15%. The percentage of mapped reads among the 60 F_1_ gametophytes was between 30.20 and 83.75%, with an average of 57.33%. The differences in the mapping rates among individuals might be caused by exogenous DNA contamination or unprovided reference genome sequences of *Gp. lemaneiformis*.

As a result, the miss rate of SNP genotypes was less than 15% in 62 gametophytes, and 46,499 SNPs were identified, representing 1.83% homozygous loci (no homozygous genotypes occurred in the 62 gametophytes) and 98.17% heterozygous loci (heterozygous genotypes occurred in at least one individual). Further statistical analyses revealed that 22,640 SNP loci had identical heterozygous genotypes in the 62 gametophytes, accounting for 48.69% of the total SNPs.

### Discarding SNPs Located in Repetitive Sequences

The annotation of repetitive sequences had been conducted previously (Zhou et al., 2013). SNP loci that were located in repetitive sequences were discarded, and 9,989 SNP markers remained. In these SNPs, the number of loci that were identified as having a heterozygous genotype in at least one individual accounted for 92.02%, and 48.07% loci had identical heterozygous genotypes in the 62 gametophytes. In addition, the number of homozygous SNPs with different genotypes in the two parents was only 25, and these loci did not have 1:1 segregation ratios in the 60 F_1_ gametophytes. Genotyping results of representative SNP loci in two parents and partial gametophytes are displayed in **Table 1**. Complete genotype information for the 9,989 SNPs is shown in **Appendix S1**.

**Table 1:** Genotyping results of representative SNP loci in two parents and partial F_1_ gametophytes using whole-genome resequencing technology.

Chr	Position	Ref	Alt	♀6	♂9	ZD10	ZD20	ZD30	ZD40	ZD50	ZD60
Scaffold32	7259^a^	T	A	W	W	W	W	W	W	W	W
Scaffold19	12935^b^	A	G	A	R	A	R	R	R	–	R
Scaffold42	123^c^	C	T	T	T	T	T	T	T	T	T
Scaffold311	1271^d^	T	C	T	C	C	C	C	C	C	C
Scaffold1438	26108^e^	T	G	T	G	T	T	T	K	K	G

Ref indicates the SNP genotype was in accordance with that of the reference genome sequence; Alt indicates that the SNP genotype was inconsistent with that of the reference genome sequence; ZD X indicates No. X F_1_ gametophyte; W = A/T; R = A/G; K = G/T; ^a^The SNP loci that had identical heterozygous genotypes in 62 gametophytes accounted for 48.07% of the total SNPs; ^b^The SNP loci that had a homozygous (or missing) genotype in one parent and a heterozygous genotype in the other accounted for 17.06% of the total SNPs; ^c^The SNP loci that had identical homozygous genotypes, but inconsistent with Ref, in the 62 gametophytes accounted for 7.43% of the total SNPs; ^d^The SNP loci that had homozygous genotypes in the two parents was but only one homozygous genotype in the F_1_ gametophytes accounted for 0.12% of the total SNPs; ^e^The SNP loci that had homozygous genotypes in both parents but were heterozygous in some F_1_ gametophytes accounted for 0.13% of the total SNPs.

**Figure 1:**
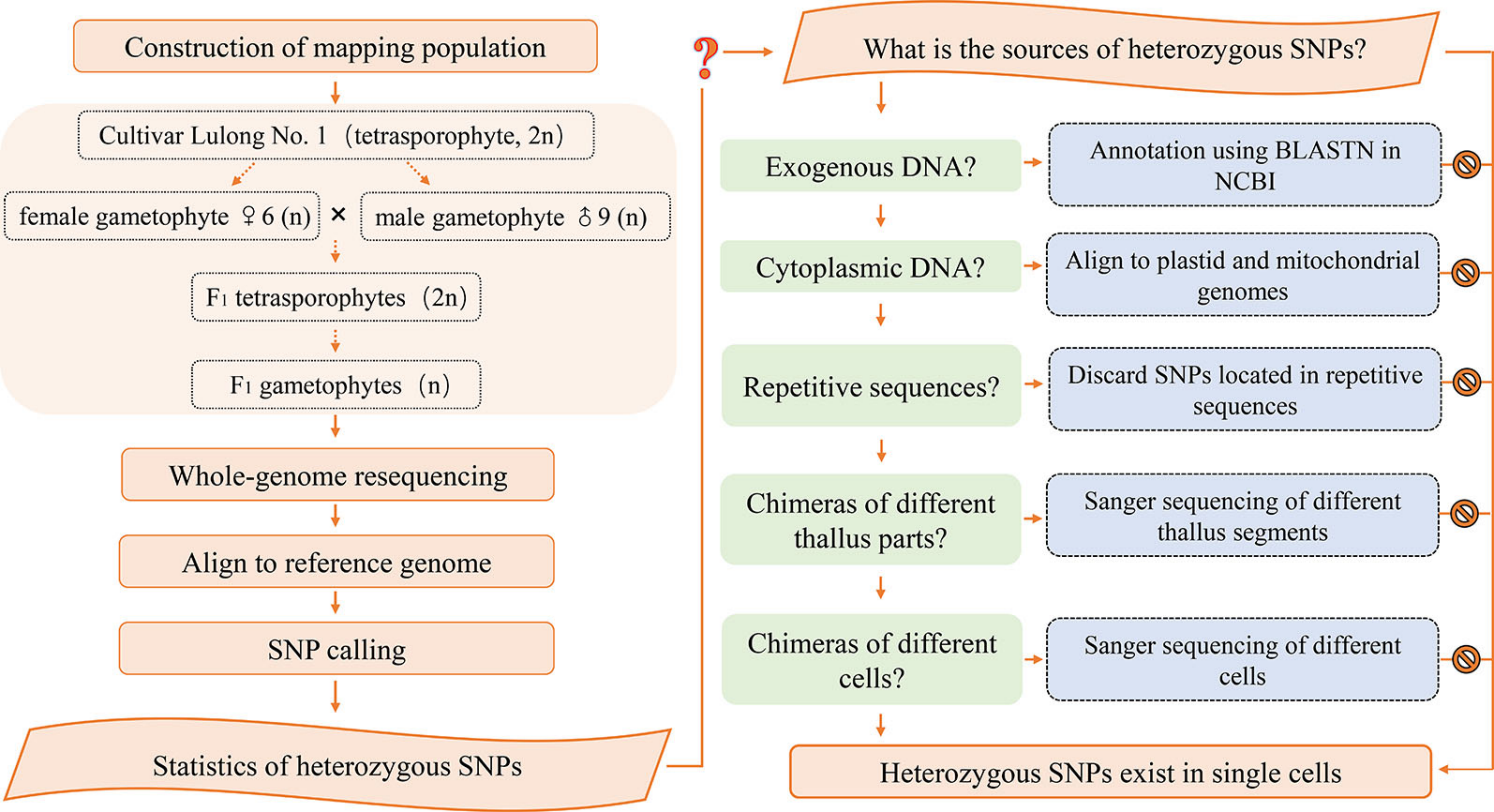
Flowchart of the research methodology.

For each gametophyte, the proportions of homozygous and heterozygous loci ranged between 13.74 and 21.61% (mean of 17.04%) and between 66.36 and 83.59% (mean of 77.12%), respectively. The remainder were missing loci, representing an average 5.84% (**Figure 1**). An overwhelming number of heterozygous loci existed in all the gametophytes.

### Verification of Heterozygous SNPs

The unmapped reads obtained from whole-genome resequencing were removed to filter out exogenous DNA contamination. However, because the reference genome was the result of a preliminary genome survey assembly, it was possible that exogenous DNA existed in the assembled reference genome. The GC content and average sequencing depth of the genomic data of *Gp. lemaneiformis* had been reported previously (Zhou et al., 2013) and are shown in **Figure S3**. The density points were concentrated in the 40–60% range, revealing no exogenous DNA contamination. For further verification, 6,755 SNPs that had identical heterozygous genotypes in the two parental gametophytes were annotated using BLASTN in NCBI. As shown in **Table 2**, 6,687 sequences were not annotated to any species, representing a proportion of 98.99%. In total, 68 sequences were mapped to similar sequences in the NT database, with matched species from four classes, bacteria, fungi, red alga, and animal, accounting for only 1%. The annotation results reflected the unlikelihood that the heterozygous SNPs were derived from exogenous DNA.

**Table 2:** Annotation results of the sequences containing heterozygous SNP loci in *Gracilariopsis lemaneiformis*.

Species category	Species name	Number of sequences
Bacteria	*Burkholderia multivorans*	5
	*Mixia osmundae*	9
	*Scedosporium apiospermum*	1
	total	15(0.22%)
Fungus	*Branchiostoma belcheri*	4
	*Saccharomyces cerevisiae*	4
	*Naumovozyma dairenensis*	3
	total	11(0.16%)
Rhodophyta	*Chondrus crispus*	15
	*Furcellaria lumbricalis*	2
	*Gracilaria chorda*	2
	*Gracilaria gracilis*	3
	*Gracilaria vermiculophylla*	2
	*Gracilariopsis lemaneiformis*	3
	total	27(0.40%)
Zoology	*Mus musculus*	1
	*Octodon degus*	5
	*Spodoptera frugiperda*	9
	total	15(0.22%)
Unknown		6687(98.99%)
Total		6755(100%)

**Figure 2:**
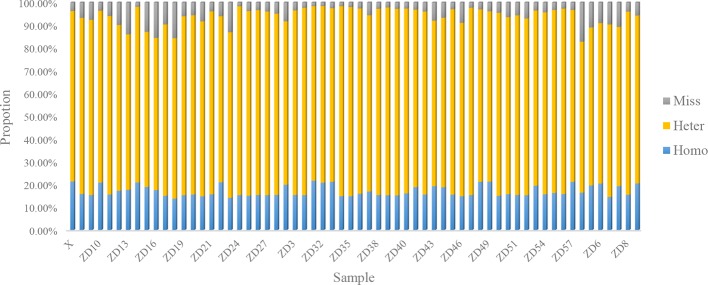
Statistics of SNP type in parents and 60 gametophytes.

In this study, each gametophyte was cultivated in a separate bottle and the genomic DNA was extracted independently, which should eliminate the possibility of cross contamination. Moreover, a high proportion of heterozygous sites existed in each individual, as shown in **Figure 2**, which further indicated that the heterozygous SNPs did not result from cross contamination among certain individuals.

In total, 1,704 SNP loci that had homozygous (or missing) genotypes in one parent, but heterozygous genotypes in the other parent, were aligned to the complete plastid and mitochondrial sequences of *Gp. lemaneiformis*. However, none of the sequences containing SNPs were mapped to plastid or mitochondrial sequences, excluding the possibility that sequence contamination occurred from plastids or mitochondria.

**Figure 3:**
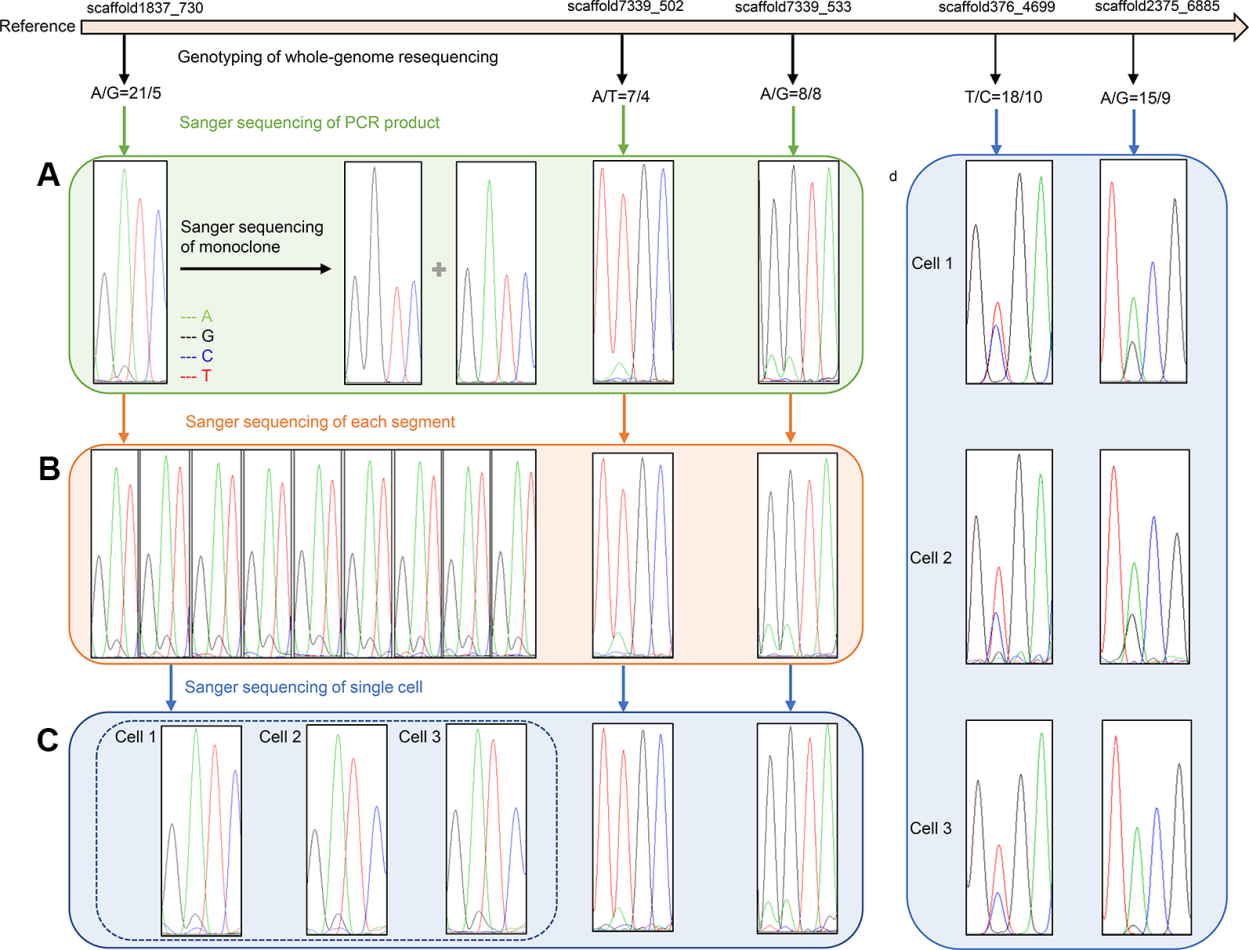
Verification results of heterozygous SNPs **(A)** Sanger sequencing results of three SNP loci (scaffold1837_730, scaffold7339_502 and scaffold7339_533) in one F_1_ gametophyte. Two peaks of each SNP cite are consistent with the genotyping results of whole-genome resequencing. Monoclonal sequencing results of the locus scaffold1837_730 show both single peak A and single peak G, which completely verified the accuracy of the genotyping results A/G. **(B)** Sanger sequencing results of three SNP loci in different segments of one F_1_ gametophyte. The Sanger sequencing results of nine representative parts at locus scaffold1837_730 are completely coincident, showing two peak A and G, and are consistent with genotyping results A/G. **(C)** Sanger sequencing results of three SNP loci in three cells of one F_1_ gametophytes. The Sanger sequencing results of three cells at locus scaffold1837_730 are completely coincident, showing two peaks A and G, and are consistent with the genotyping results A/G. **(D)** Sanger sequencing results of three cells at two SNP sites (scaffold376_4699 and scaffold2375_6885) located in gene regions. Two Sanger sequencing peaks of each SNP site located in gene region still existed, which are consistent with the genotyping results.

Information regarding primers that were used to amplify SNP loci are listed in **Table 3**. Sanger sequencing results for three SNP loci in one F_1_ gametophyte are displayed in **Figure 3A**. The genotyping results of the SNP locus scaffold1837_730 was A/G, and the Sanger sequencing of PCR products showed two peaks in this site, with peak A being higher than peak G. In addition, Sanger sequencing results of SNP loci scaffold7339_502 and scaffold7339_533 in this gametophyte also showed two peaks of SNPs that were consistent with the genotyping results obtained from whole-genome resequencing. To demonstrate the existence of the lower peak, monoclonal sequencing of the locus scaffold1837_730 was carried out in this gametophyte. In total, 54 monoclones were selected for sequencing. Of these, 53 monoclones had the single peak A and one monoclone had the single peak G, which verified the accuracy of the genotyping results.

**Table 3:** PCR primers used for Sanger sequencing.

ID	Chr	Position of SNP	Ref	Alt	Sense primer	Anti-sense primer	Product length (bp)
P1	scaffold1837	730	A	G	TCCTCCTTACGGCGGTTGAAGTT	TGGATACCGACTCGGACGAATACG	301
P2	scaffold7339	502/533	A	T	AGCAGAAGACGGAGCGAACGATGA	AGTTGCAGCAATCCGGCCAATAGG	322
P3	scaffold376	4699	T	C	ATGTCTTCGTACTTCGCTCCT	TACGGCGGTGAAACCGACTT	485
P4	scaffold2375	6885	G	A	CCTATGTCACCCGATTGTTGT	ATGCAAGAGCGACTTGACGA	757

**Table 4:** Genotyping results of 3 SNP loci in F_1_ gametophytes (No.13) using whole-genome resequencing technology and Sanger sequencing results in different segments and single cells.

ID of SNP loci	F_1_ gametophyte (No.13)	9 representative segments of F_1_ gametophyte (No.13)									3 cells of F_1_ gametophyte (No.13)		
		1	2	3	4	5	6	7	8	9	1	2	3
Scaffold 1837_730	A/G	A/G	A/G	A/G	A/G	A/G	A/G	A/G	A/G	A/G	A/G	A/G	A/G
Scaffold 7339_502	A/T	A/T	A/T	A/T	A/T	A/T	A/T	A/T	A/T	A/T	A/T	A/T	A/T
Scaffold 7339_533	A/G	A/G	A/G	A/G	A/G	A/G	A/G	A/G	A/G	A/G	A/G	A/G	A/G

### Excluding the Possibility of Chimerism

All 22 segments that were cut from one gametophyte were amplified using primers for SNP locus scaffold1837_730. The Sanger sequencing results of each segment were completely coincident, showing two peaks, A and G, and even the height differences between peaks A and G were identical (**Figure 3B**).

Additionally, SNP loci scaffold7339_502 and scaffold7339_533 were detected in each part of the gametophyte. For each SNP locus, the Sanger sequencing results for each part were completely coincident and were consistent with the genotyping results derived from whole-genome resequencing, as shown in **Figure 3B** and **Table 4**.

### Detection of Heterozygous SNPs in a Single Cell

Single cells were isolated and selected using glass capillary tube (**Figure S4**). Sanger sequencing results for three SNP loci (scaffold1837_730s, scaffold7339_502 and scaffold7339_533) in three cells from one F_1_ gametophyte are displayed in **Figure 3C**. The results were consistent with the genotyping results, as shown in **Table 4**. Thus, heterozygous SNP sites existed in single cells.

To eliminate background interference, high fidelity DNA polymerase was used to conduct the PCR amplification of three cells. Two SNP loci, scaffold376_4699 and scaffold2375_6885, which were located in genic regions, were further used to test single cells. Sanger sequencing results revealed that the two peaks of each SNP locus located in the genomic region still existed, which was consistent with the genotyping results of the whole-genome resequencing (**Figure 3D**). Thus, the SNP genotypes were shown to be heterozygous in individual cells.

### Inference That Heterozygous SNPs Exist in Haploid Gametophytes of *Gp. lemaneiformis*

Based on our results, exogenous DNA contamination, cross contamination among individuals, plastid and mitochondrial sequences, chimeras of different thallus parts, or different cells, and repetitive sequences were excluded as being the source of heterozygous SNPs. However, the reference sequence we used to identify SNPs in this study, was a preliminary assembly version, with a scaffold N50 of 20 kb and a scaffold number of 125,685. If a chromosomal rearrangement event, for instance, a duplication and deletion, occurred in the genome of *Gp. lemaneiformis*, the length of a scaffold might not cover the large area of duplication. Consequently, the unique scaffold may actually exist in multiple copies in the genome. This could explain why heterozygous SNPs existed in haploid gametophyte genomes.

## Discussion

In this study, whole-genome resequencing was conducted to develop SNP markers, which have been widely applied in many plants. Whole-genome resequencing of two parental lines *Brassica oleracea* L. C1184 and C1234 was performed to develop genome-wide SNP markers (Lee et al., 2015), and the sequencing data represented approximately 18-fold genome coverage for both parental lines. After reference-based genome-guided mapping, 82.1 and 77.6% of reads from C1184 and C1234, respectively, were successfully aligned to the reference genome. As a result, a total of 674,521 SNPs was detected between C1184 and C1234. In *Capsicum baccatum* L. (Lee et al., 2016), the parental genomes were sequenced using the whole-genome resequencing method. After trimming the raw data, the parental sequences corresponded to approximately 3.1 and 9.6 times, respectively, the size of the reference genome. Then, 48.5% of the trimmed reads were successfully mapped to the reference sequences. Finally, 97,085 SNPs were selected as homozygous SNPs between the parents. Whole-genome resequencing was performed on 96 F_6_ recombinant inbred lines (RILs) of a cross between safflower *Carthamus tinctorius* L. and its wild progenitor *C. palaestinus* Eig (Bowers et al., 2016). Each RIL was sequenced to an average 2.26 × coverage of the estimated genome, while the parents were sequenced to a greater depth. Sequence reads from the RILs were mapped to the reference genome, and 2,008,196 SNPs were identified.

In our result, the number of SNPs (46,499) identified by whole-genome resequencing in *Gp. lemaneiformis* was smaller than in other plants, which might result from the small genome and population sizes. However, as C-value paradox indicates that there is no absolute correlation between the size of a genome and evolutionary status of an organism. Although the genome size of *Gp. lemaneiformis* is only 97.02 Mb (Zhou et al., 2013), its complexity level may be high.

The reference genome sequences selected in this study were from the first genome version, which consisted of 125,685 scaffolds with an N50 length of 20 kb (Zhou et al., 2013) and not from the second version, which consisted of 13,825 scaffolds with an N50 length of 30.59 kb (Sun et al., 2018). The main reason for the selection was that first genome sequencing was a wild female gametophyte, as our resequencing gametophyte, while in the second version, the material was the 981 strain, which is a diploid tetrasporophyte.

SNP markers were first developed in F_1_ gametophytes of *Gp. lemaneiformis* using whole-genome resequencing technology, laying a foundation for construction of genetic linkage map and localization of quantitative trait locus. We further identified 2,386 SNPs which were located in 818 genes. For example, SNPs (scaffold2671_ 31163,31176,31189,31266) were located in the gene region of *LEM_GLEAN_10003235* whose function was annotated as carotenoid isomerase, which is an important enzyme in synthesis pathway of carotenoids. Moreover, *LEM_GLEAN_0001204*, the gene encoding phosphoglucose isomerase in metabolic pathways of agar, contains one SNP locus (scaffold1981_ 16188). These SNPs could be potentially used as genetic markers for the selection and provided the theoretical basis of molecular marker assisted breeding

An overwhelming number of heterozygous SNP loci were verified to exist in the 62 gametophytes, which might result from chromosomal duplication events in the genome of *Gp. lemaneiformis*. There are several kinds of chromosomal rearrangement, including the duplication or deletion of a chromosomal segment, the inversion of a segment and the translocation of segments between non-homologous chromosomes (Rieseberg 2001). Each chromosomal rearrangement event can be caused by the breakage of DNA double-helices at two different locations, followed by rejoining or by crossing-over between repetitive DNA (Griffiths et al., 1999). Chromosomal rearrangements in the genome can produce duplicated genes, which may differ in DNA sequence, gene structure and even function. One genetic effect of duplication is that the gene expression may be enhanced as the gene copies increase in number.

A chromosome level of assembly is required to verify the source of the heterozygous SNPs. This study promoted the assembly of a *Gp. lemaneiformis* genomic map of using single molecule real-time sequencing (SMRT), or high-throughput chromosome conformation capture. SMRT sequencing can produce considerably longer and highly accurate reads from single unamplified molecules. The average read length of PacBio RS II platform is about 15 kb. The long SMRT sequencing reads will span complex repeats and missing bases, which will substantially improve genome complete assembly. A complete genome has the potential to facilitate genome-assisted breeding and will be useful to investigate genomic patterns in the evolution of marine algae.

## Data Availability Statement

The datasets generated for this study can be found in the Short Read Archive (SRA) database with Project number PRJNA574029, under the accession number SRP223151 (SRR10177194 – SRR10177255).

## Author Contributions

ZS and YH contributed conception and design of the study. YH, WZho and JW constructed the mapping population. YH performed the experiments. YH and ZS analyzed the data. MJ, HC, JZ, WZha, XF, YL, BX and PG contributed reagents/materials/analysis tools. YH wrote the manuscript and ZS made revision. All authors read and approved the submitted version.

## Funding

This work was supported by the China Agriculture Research System (CARS-50), the National Natural Science Foundation of China (No. 31372529) and the Fundamental Research Funds for the Central Universities (No. 201762016).

## Conflict of Interest

The authors declare that the research was conducted in the absence of any commercial or financial relationships that could be construed as a potential conflict of interest.
