# Supplementary material for: Heterozygous Single Nucleotide Polymorphic Loci in Haploid Gametophytes of Gracilariopsis lemaneiformis (Rhodophyta)
Source: Front Genet. 2019 Dec 6;10:1256. doi: 10.3389/fgene.2019.01256 (PMC6915112; doi:10.3389/fgene.2019.01256)
Supplement: Supplementary file 2 [file DataSheet_2.docx]

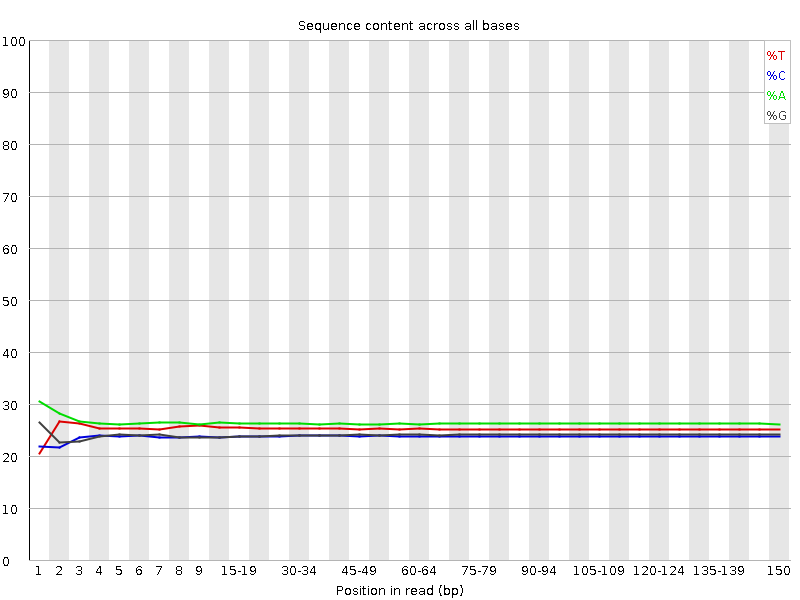


**Figure S1 Base distribution for whole-genome resequencing data.**


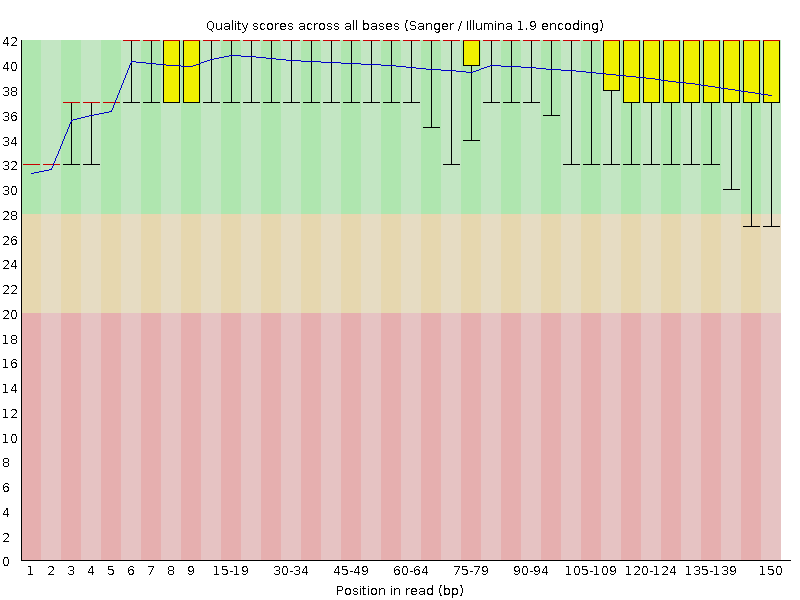


**Figure S2 Base quality distribution for whole-genome resequencing data.**


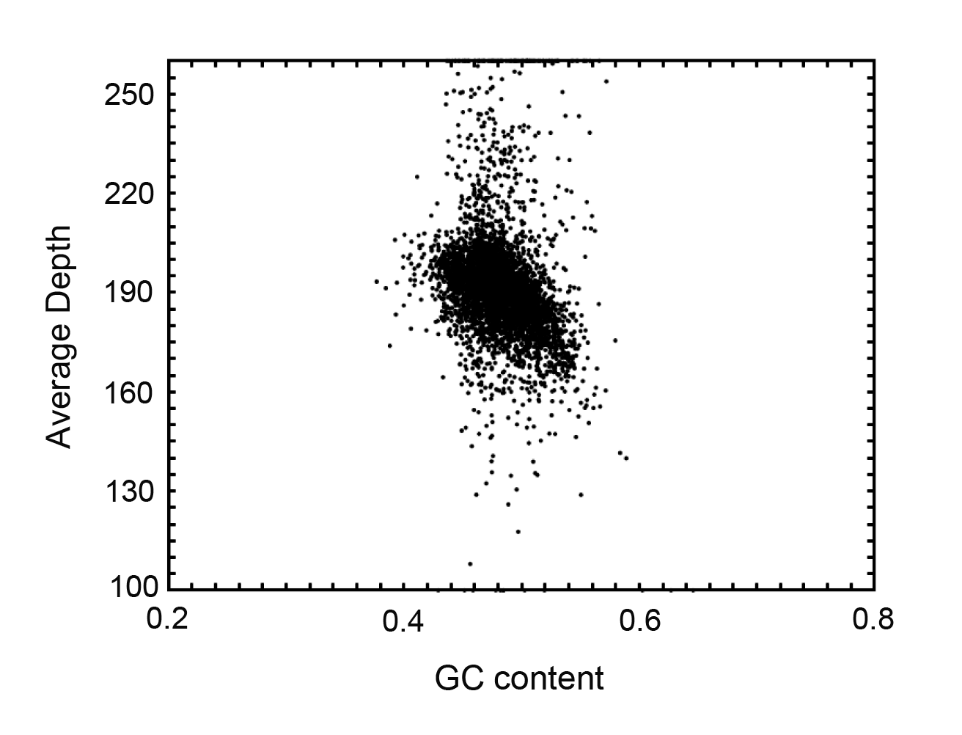


**Figure S3 GC content and average sequencing depth of the genome data of *Gp. lemaneiformis* (Zhou et al. 2013).**

Zhou, W., Hu, Y. Y., Sui, Z. H., Fu, F., Wang, J. G., Chang, L. P., Guo, W. H., Li, B. B. 2013. Genome Survey Sequencing and Genetic Background Characterization of *Gracilariopsis lemaneiformis* (Rhodophyta) Based on Next-Generation Sequencing. *PloS One* **8**:e69909.


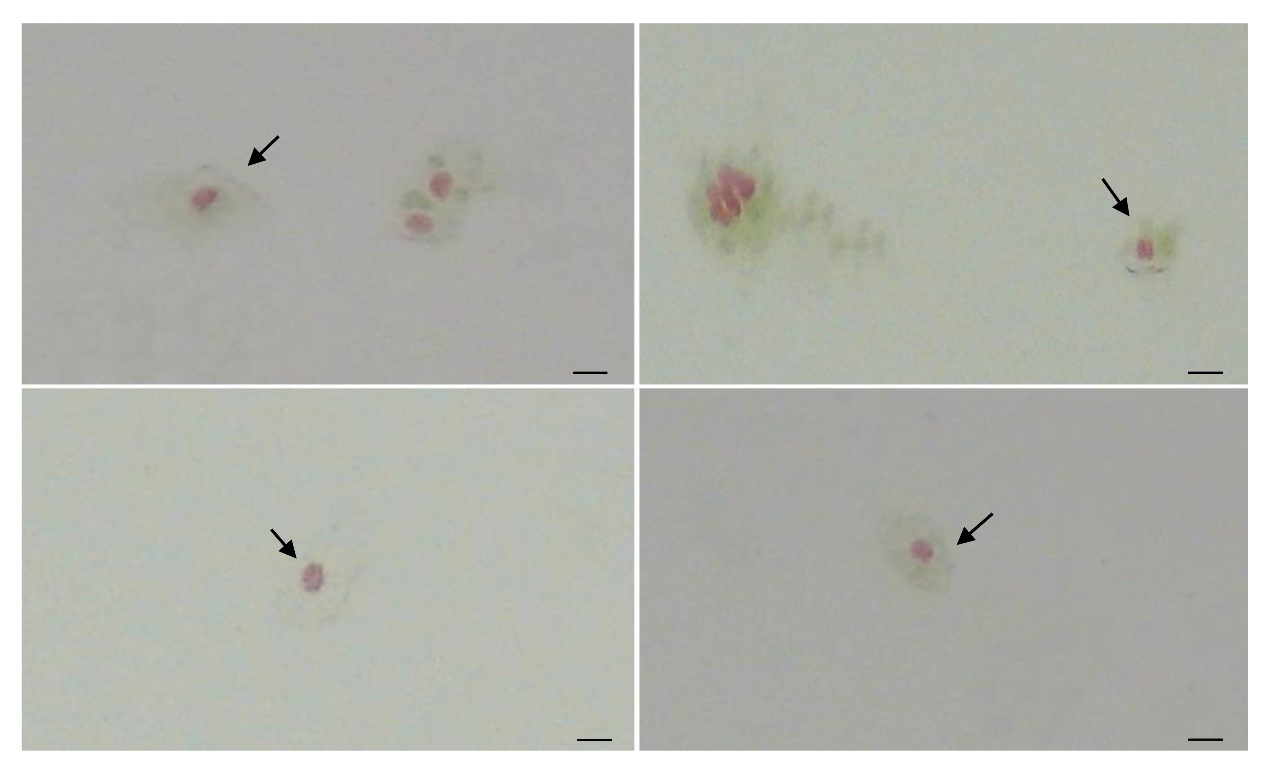


**Figure S4 Single cell of gametophyte selected by capillary glass tube (Scale bar: 15 μm)**
