# Supplementary material for: Heterozygous Single Nucleotide Polymorphic Loci in Haploid Gametophytes of Gracilariopsis lemaneiformis (Rhodophyta)
Source: Front Genet. 2019 Dec 6;10:1256. doi: 10.3389/fgene.2019.01256 (PMC6915112; doi:10.3389/fgene.2019.01256)
Supplement: Supplementary file 3 [file DataSheet_3.docx]

**Table S1** Data statistics of whole-genome resequencing of *Gracilariopsis lemaneiformis*

| Sample | Total_Bases | Error% | Q20% | Q30% | GC% | Depth× | Coverage |
| --- | --- | --- | --- | --- | --- | --- | --- |
| ♀6 | 1.21E+09 | 0.0118 | 98.00 | 94.20 | 48.09 | 12 | 80.55% |
| ♂9 | 1.36E+09 | 0.0124 | 97.80 | 93.61 | 50.73 | 14 | 79.30% |
| ZD1 | 1.90E+09 | 0.0115 | 97.23 | 93.58 | 52.53 | 19 | 79.79% |
| ZD2 | 1.83E+09 | 0.0120 | 96.80 | 93.06 | 51.09 | 18 | 79.88% |
| ZD3 | 1.61E+09 | 0.0120 | 97.96 | 94.02 | 51.54 | 16 | 80.52% |
| ZD4 | 1.79E+09 | 0.0118 | 98.00 | 94.15 | 50.69 | 18 | 80.44% |
| ZD5 | 1.68E+09 | 0.0132 | 97.56 | 92.90 | 51.29 | 17 | 80.25% |
| ZD6 | 1.26E+09 | 0.0120 | 97.94 | 94.05 | 47.88 | 13 | 80.24% |
| ZD7 | 1.57E+09 | 0.0120 | 97.93 | 94.00 | 49.42 | 16 | 79.76% |
| ZD8 | 1.70E+09 | 0.0120 | 97.92 | 93.98 | 48.11 | 17 | 80.34% |
| ZD9 | 1.60E+09 | 0.0122 | 97.86 | 93.80 | 51.52 | 16 | 80.17% |
| ZD10 | 1.86E+09 | 0.0127 | 97.65 | 93.29 | 49.10 | 19 | 80.62% |
| ZD11 | 1.56E+09 | 0.0121 | 97.88 | 93.87 | 49.76 | 16 | 79.85% |
| ZD12 | 1.86E+09 | 0.0116 | 97.07 | 93.46 | 52.33 | 19 | 79.50% |
| ZD13 | 1.46E+09 | 0.0120 | 97.96 | 94.02 | 51.07 | 15 | 78.75% |
| ZD14 | 1.50E+09 | 0.0128 | 96.17 | 91.98 | 48.21 | 15 | 80.94% |
| ZD15 | 1.31E+09 | 0.0116 | 97.14 | 93.40 | 51.63 | 13 | 78.92% |
| ZD16 | 1.22E+09 | 0.0114 | 97.15 | 93.67 | 51.46 | 12 | 78.74% |
| ZD17 | 1.81E+09 | 0.0113 | 97.14 | 93.79 | 46.71 | 18 | 79.23% |
| ZD18 | 1.40E+09 | 0.0123 | 96.45 | 92.61 | 49.06 | 14 | 78.02% |
| ZD19 | 1.43E+09 | 0.0112 | 97.27 | 93.97 | 46.29 | 14 | 79.87% |
| ZD20 | 1.44E+09 | 0.0124 | 96.39 | 92.53 | 47.52 | 14 | 79.16% |
| ZD21 | 1.30E+09 | 0.0116 | 96.99 | 93.47 | 50.40 | 13 | 80.71% |
| ZD22 | 1.81E+09 | 0.0129 | 96.21 | 91.93 | 49.81 | 18 | 80.19% |
| ZD23 | 1.48E+09 | 0.0114 | 97.22 | 93.61 | 49.72 | 15 | 78.77% |
| ZD24 | 2.04E+09 | 0.0114 | 97.11 | 93.56 | 47.71 | 20 | 81.13% |
| ZD25 | 1.62E+09 | 0.0115 | 97.13 | 93.50 | 47.15 | 16 | 80.94% |
| ZD26 | 1.73E+09 | 0.0112 | 97.27 | 93.91 | 45.88 | 17 | 80.08% |
| ZD27 | 1.45E+09 | 0.0119 | 96.87 | 93.09 | 48.00 | 15 | 80.61% |
| ZD28 | 1.84E+09 | 0.0112 | 97.24 | 93.96 | 48.71 | 18 | 79.94% |
| ZD29 | 1.40E+09 | 0.0118 | 96.68 | 93.07 | 47.73 | 14 | 80.38% |
| ZD30 | 1.68E+09 | 0.0114 | 97.02 | 93.72 | 47.42 | 17 | 80.82% |
| ZD31 | 1.59E+09 | 0.0127 | 96.12 | 92.15 | 47.30 | 16 | 81.09% |
| ZD32 | 1.73E+09 | 0.0114 | 97.10 | 93.82 | 47.07 | 17 | 80.92% |
| ZD33 | 1.48E+09 | 0.0119 | 96.76 | 93.13 | 47.79 | 15 | 81.16% |
| ZD34 | 1.59E+09 | 0.0114 | 97.18 | 93.74 | 49.76 | 16 | 80.89% |
| ZD35 | 1.69E+09 | 0.0117 | 96.73 | 93.23 | 46.97 | 17 | 81.14% |
| ZD36 | 1.60E+09 | 0.0119 | 96.34 | 93.08 | 46.04 | 16 | 81.24% |
| ZD37 | 1.33E+09 | 0.0111 | 97.28 | 94.07 | 47.24 | 13 | 80.85% |
| ZD38 | 1.47E+09 | 0.0114 | 97.03 | 93.72 | 49.63 | 15 | 80.36% |
| ZD39 | 1.60E+09 | 0.0109 | 97.45 | 94.37 | 45.03 | 16 | 81.02% |
| ZD40 | 1.55E+09 | 0.0110 | 97.45 | 94.08 | 45.68 | 16 | 81.17% |
| ZD41 | 1.74E+09 | 0.0116 | 97.09 | 93.46 | 51.35 | 17 | 81.29% |
| ZD42 | 1.86E+09 | 0.0117 | 96.86 | 93.26 | 46.80 | 19 | 79.81% |
| ZD43 | 1.73E+09 | 0.0116 | 97.02 | 93.39 | 45.55 | 17 | 79.61% |
| ZD44 | 1.63E+09 | 0.0115 | 96.95 | 93.56 | 46.55 | 16 | 79.80% |
| ZD45 | 2.03E+09 | 0.0112 | 97.26 | 93.91 | 49.51 | 20 | 80.43% |
| ZD46 | 1.74E+09 | 0.0115 | 97.01 | 93.56 | 47.05 | 17 | 79.10% |
| ZD47 | 1.62E+09 | 0.0118 | 96.82 | 93.15 | 49.00 | 16 | 80.87% |
| ZD48 | 1.47E+09 | 0.0111 | 97.34 | 94.11 | 50.30 | 15 | 80.62% |
| ZD49 | 1.62E+09 | 0.0117 | 96.97 | 93.21 | 48.95 | 16 | 80.25% |
| ZD50 | 1.64E+09 | 0.0119 | 96.80 | 93.03 | 50.82 | 16 | 79.96% |
| ZD51 | 1.59E+09 | 0.0111 | 97.25 | 94.04 | 46.99 | 16 | 79.76% |
| ZD52 | 2.00E+09 | 0.0115 | 96.98 | 93.52 | 48.63 | 20 | 79.35% |
| ZD53 | 1.79E+09 | 0.0116 | 97.00 | 93.41 | 49.84 | 18 | 80.89% |
| ZD54 | 1.64E+09 | 0.0133 | 95.10 | 91.58 | 50.05 | 16 | 80.10% |
| ZD55 | 1.62E+09 | 0.0121 | 96.58 | 92.82 | 48.84 | 16 | 80.92% |
| ZD56 | 1.62E+09 | 0.0134 | 95.82 | 91.41 | 50.01 | 16 | 80.46% |
| ZD57 | 1.82E+09 | 0.0114 | 97.19 | 93.84 | 51.34 | 18 | 80.84% |
| ZD58 | 1.56E+09 | 0.0124 | 96.54 | 92.48 | 51.32 | 16 | 78.36% |
| ZD59 | 1.65E+09 | 0.0117 | 97.03 | 93.26 | 48.54 | 17 | 79.38% |
| ZD60 | 1.91E+09 | 0.0113 | 97.26 | 93.85 | 50.64 | 19 | 79.00% |

**Table S2** Mapping rate statistics of whole-genome resequencing of *Gracilariopsis lemaneiformis*

| Sample | All_Mapped_Reads | Paired_Mapping_Reads | Single_Mapping_Reads |
| --- | --- | --- | --- |
| ♀6 | 6196370(84.35%) | 5151043(70.12%) | 259509(3.53%) |
| ♂9 | 4171234(49.15%) | 3502716(41.27%) | 176979(2.09%) |
| ZD1 | 4060202(35.91%) | 3351995(29.64%) | 204844(1.81%) |
| ZD2 | 4770237(43.18%) | 3994388(36.15%) | 222114(2.01%) |
| ZD3 | 5847277(62.15%) | 4785638(50.86%) | 295193(3.14%) |
| ZD4 | 6263129(59.23%) | 5208378(49.25%) | 311016(2.94%) |
| ZD5 | 5331093(52.66%) | 4421838(43.67%) | 284151(2.81%) |
| ZD6 | 4268854(59.46%) | 3564645(49.65%) | 201082(2.80%) |
| ZD7 | 3861551(42.05%) | 3237463(35.25%) | 195150(2.13%) |
| ZD8 | 5739532(58.16%) | 4651378(47.13%) | 348000(3.53%) |
| ZD9 | 4769305(49.45%) | 3968749(41.15%) | 233544(2.42%) |
| ZD10 | 5618333(50.48%) | 4725384(42.46%) | 234330(2.11%) |
| ZD11 | 4982776(51.00%) | 4144504(42.42%) | 219206(2.24%) |
| ZD12 | 3913403(33.21%) | 3284545(27.87%) | 177707(1.51%) |
| ZD13 | 3000572(32.26%) | 2549169(27.41%) | 124612(1.34%) |
| ZD14 | 7562876(82.81%) | 6332632(69.34%) | 290863(3.18%) |
| ZD15 | 3267493(39.44%) | 2725919(32.91%) | 156088(1.88%) |
| ZD16 | 3128950(40.58%) | 2608233(33.82%) | 150986(1.96%) |
| ZD17 | 3597144(31.39%) | 2974973(25.96%) | 181469(1.58%) |
| ZD18 | 2820322(31.73%) | 2317069(26.07%) | 161151(1.81%) |
| ZD19 | 5028249(56.55%) | 4095648(46.06%) | 267196(3.01%) |
| ZD20 | 3871257(42.59%) | 3250091(35.76%) | 174282(1.92%) |
| ZD21 | 5978362(81.01%) | 4905344(66.47%) | 267695(3.63%) |
| ZD22 | 4359229(40.51%) | 3688049(34.28%) | 189003(1.76%) |
| ZD23 | 3161539(34.87%) | 2617839(28.87%) | 188128(2.07%) |
| ZD24 | 7983032(67.15%) | 6672908(56.13%) | 411715(3.46%) |
| ZD25 | 6520782(69.43%) | 5333250(56.78%) | 333422(3.55%) |
| ZD26 | 6009180(58.77%) | 5114810(50.03%) | 287749(2.81%) |
| ZD27 | 6270463(74.89%) | 5062697(60.47%) | 344372(4.11%) |
| ZD28 | 5082487(45.82%) | 4232335(38.16%) | 269615(2.43%) |
| ZD29 | 4403429(54.77%) | 3636066(45.23%) | 235037(2.92%) |
| ZD30 | 6911017(71.79%) | 5889056(61.18%) | 295152(3.07%) |
| ZD31 | 7601970(83.75%) | 6414467(70.67%) | 321424(3.54%) |
| ZD32 | 7933536(79.22%) | 6783205(67.73%) | 342940(3.42%) |
| ZD33 | 7025923(83.35%) | 5883492(69.80%) | 294633(3.50%) |
| ZD34 | 7667905(82.43%) | 6498956(69.87%) | 276744(2.98%) |
| ZD35 | 7668963(80.50%) | 6427580(67.47%) | 302137(3.17%) |
| ZD36 | 7479116(80.72%) | 6240720(67.35%) | 291478(3.15%) |
| ZD37 | 5953051(78.29%) | 4856366(63.87%) | 307279(4.04%) |
| ZD38 | 6569362(76.31%) | 5549299(64.46%) | 283691(3.30%) |
| ZD39 | 7270693(78.53%) | 6121104(66.11%) | 308124(3.33%) |
| ZD40 | 7002127(78.09%) | 5869285(65.45%) | 314926(3.51%) |
| ZD41 | 7810035(79.15%) | 6535240(66.23%) | 326952(3.31%) |
| ZD42 | 5340216(48.04%) | 4537260(40.81%) | 254590(2.29%) |
| ZD43 | 4165604(39.98%) | 3581547(34.37%) | 150180(1.44%) |
| ZD44 | 5228241(54.19%) | 4461372(46.24%) | 268687(2.79%) |
| ZD45 | 6932434(58.97%) | 5842417(49.70%) | 357764(3.04%) |
| ZD46 | 3672601(35.28%) | 3088689(29.67%) | 172411(1.66%) |
| ZD47 | 7086032(75.29%) | 5969604(63.43%) | 331741(3.52%) |
| ZD48 | 6508260(77.51%) | 5548597(66.08%) | 295610(3.52%) |
| ZD49 | 5849191(61.39%) | 4998250(52.46%) | 274238(2.88%) |
| ZD50 | 5003549(51.34%) | 4209883(43.19%) | 266449(2.73%) |
| ZD51 | 4806449(51.84%) | 4068930(43.89%) | 214294(2.31%) |
| ZD52 | 3992266(32.84%) | 3392768(27.91%) | 166589(1.37%) |
| ZD53 | 6291770(60.58%) | 5346652(51.48%) | 248777(2.40%) |
| ZD54 | 5192769(54.37%) | 4377818(45.84%) | 214286(2.24%) |
| ZD55 | 6928100(73.21%) | 5808261(61.38%) | 295968(3.13%) |
| ZD56 | 6614723(69.65%) | 5567428(58.63%) | 279810(2.95%) |
| ZD57 | 6534814(62.40%) | 5572265(53.21%) | 249742(2.38%) |
| ZD58 | 2843123(31.58%) | 2409155(26.76%) | 117627(1.31%) |
| ZD59 | 3629890(37.43%) | 3100172(31.97%) | 144942(1.49%) |
| ZD60 | 3523202(30.20%) | 2912881(24.97%) | 169508(1.45%) |
